# Establishing a Musculoskeletal Oncology Unit: Teamwork Triumphs!!!

**DOI:** 10.1007/s13193-025-02198-8

**Published:** 2025-06-03

**Authors:** Manit K. Gundavda, Sahil Sood, Ashish Gulia

**Affiliations:** 1 Department of Orthopaedic Oncology, Centre for Cancer/Bone & Joint, Kokilaben Dhirubhai Ambani Hospital and Medical Research Institute, Mumbai, India; 2 Department of Radiation Oncology, Homi Bhabha Cancer Hospital and Research Centre, New Chandigarh, Punjab India; 3 Homi Bhabha Cancer Hospital and Research Centre, New Chandigarh, Punjab India

**Keywords:** Orthopaedic oncology, Clinical care, Musculoskeletal oncology

## Abstract

The establishment of a dedicated Musculoskeletal Oncology Unit within a healthcare system requires meticulous planning, coordination, and collaboration among diverse medical professionals. A multidisciplinary team approach is essential for delivering comprehensive, patient-centered care to individuals diagnosed with musculoskeletal malignancies. Integrating orthopaedic oncology into existing oncology services enhances clinical care, addressing the needs of patients with musculoskeletal tumours and providing supportive/palliative care for skeletal lesions from other primary tumours. Core members of a successful unit typically include orthopaedic surgeons, medical and radiation oncologists, radiologists, pathologists, physical therapists, and specialized nursing staff. Additionally, integrating supportive disciplines such as nutritionists, social workers, and palliative care specialists is crucial for addressing the multifaceted needs of patients. Reconstruction and rehabilitation are vital for restoring patient function. Establishing an MSK Oncology Unit within an existing oncology framework optimizes the use of shared diagnostic services like radiology, onco-pathology, nuclear imaging, and therapeutic services, providing a cost-effective model. The Department of Orthopaedic Oncology requires team members who can provide care in the operating theatre, surgical ward, and emergency departments. Given the complexity of bone and soft tissue tumour surgeries, access to surgical intensive care and blood bank support is essential. Coordinating diverse professionals presents challenges due to differing expertise and priorities, but a cohesive multidisciplinary team can yield transformative benefits. Moreover, advancements in medical technology—such as modular operating theatres, navigation equipment, 3D imaging techniques, and custom implant manufacturing—are shaping the future of personalized care in Musculoskeletal Oncology. This article summarizes our experience in establishing a MSK Oncology Unit at a tertiary cancer care level, asserting that the concept of a free-standing orthopaedic oncologist is a myth, as the foundation of this department is rooted in teamwork.

## Introduction

Musculoskeletal malignancies, encompassing both bone and soft tissue tumours, present unique challenges in diagnosis, treatment, and overall patient management [1]. The complexity of these conditions necessitates a multidisciplinary approach that integrates various medical specialties to deliver comprehensive care [2]. Establishing a dedicated Musculoskeletal Oncology Unit (MSK Oncology Unit) within existing oncology frameworks has emerged as a strategic solution to enhance patient outcomes and streamline clinical workflows [3]. By assembling a diverse team of experts—including orthopaedic surgeons, medical and radiation oncologists, radiologists, pathologists, physical therapists, and specialized nursing staff [4]—healthcare systems can create an environment that prioritizes holistic, patient-centered care [1, 2, 5]. Furthermore, the incorporation of supportive services, such as nutrition and palliative care, addresses the multifaceted needs of patients navigating their cancer journey. Outreach clinics, specialized operating rooms, training programs, research, and public health efforts are critical components of a comprehensive orthopaedic oncology department [6]. This article summarizes our experience in establishing an MSK Oncology Unit at a tertiary cancer care facility, highlighting the benefits of a cohesive team approach and the importance of shared resources in optimizing patient care.

### Patient Flow Through the Collaborative Team

Department of MSK Oncology treats patients with benign or malignant bone and soft tissue tumours as well as metastatic disease presenting in the muscular-skeletal system.

Any surgical department will encompass the team that includes the surgeon, the surgeon’s assistant, the nursing team including the scrub nurse as well as the circulating nurse, and the anaesthesia team with the chief anaesthetist and anaesthesia assistant. Surgical treatment for bone and soft tissue tumours is a much later step for these patients as the initial and rate-limiting challenge for musculoskeletal oncology remains in the diagnosis [5, 7].

The initial team of contact for patients presenting to the Orthopaedic Oncology Department consists of the multidisciplinary unit, usually involving members from the Department of Radiology and Pathology. Patients who have been referred with a suspicion of malignancy are first evaluated by this team. A musculoskeletal radiologist can convincingly determine a bone or soft tissue tumour from its routine confounders. Confirming the benign nature of certain tumours or the need for tissue diagnosis and additional investigations is determined by this team [8, 9]. Image-guided biopsy with ultrasonography or cross-sectional imaging (CT/MRI) adds to the accuracy in obtaining tissue from the lesion under evaluation [10]. Adequacy of representative tissue is confirmed by the department of pathology, and additional testing is performed to provide definitive diagnosis by the clinical as well as imaging presentation. Any discordance between the radiology or pathology information and the clinical presentation is once again reviewed at the joint clinic (which consists of multidisciplinary team members from the surgical unit, a medical oncologist, a radiation oncologist, a radiologist, and a pathologist) for providing the final diagnosis or decision [11, 12].

**Fig. 1:**
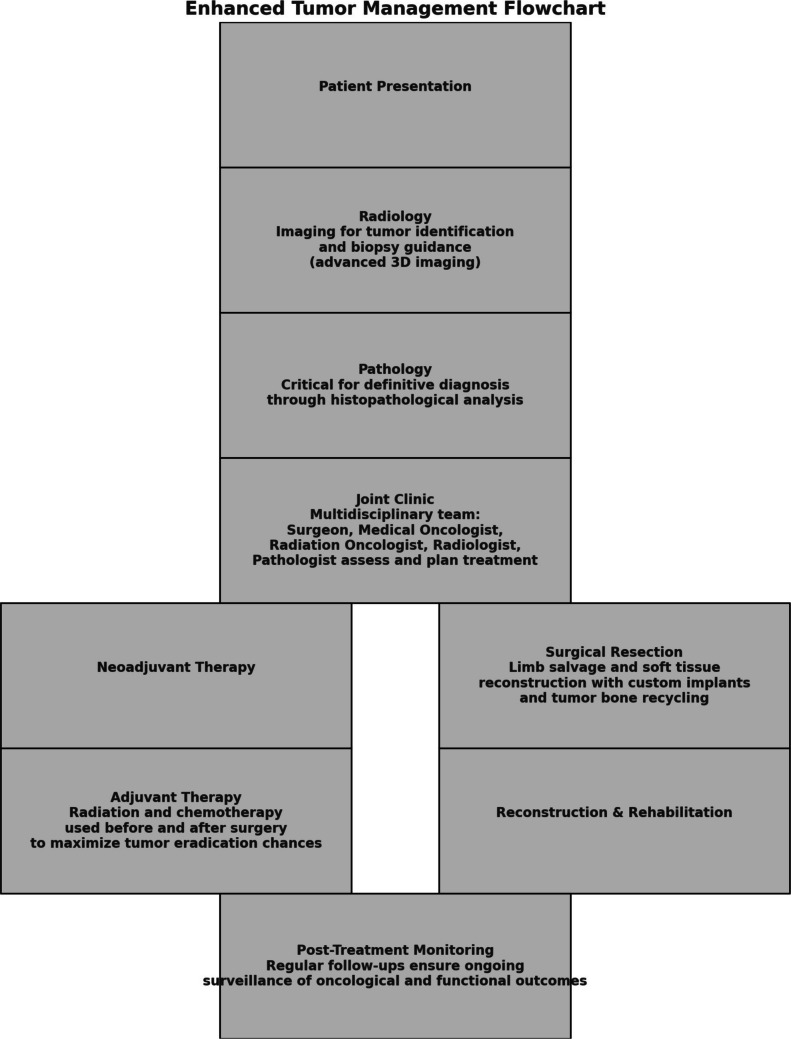
Flowchart describes the pathway for bone and soft tissue tumours requiring a multidisciplinary approach

Treatment of bone and soft tissue tumours requires a multidisciplinary approach [13]. As per the initial stage at the time of presentation, the treatment may be neoadjuvant chemotherapy with or without radiation therapy or may be upfront surgery in case of small tumours. Primary bone sarcomas such as osteosarcoma or Ewing’s sarcoma are routinely offered induction chemotherapy as per international treatment protocols under the care of the Department of Medical Oncology. Similarly, soft tissue sarcomas may be initially under the care of the Radiation Oncology Department for pre-operative radiation therapy to aid in the surgical resection of tumours. This journey of diagnosis to neoadjuvant therapy may be from a few weeks to months before any surgical intervention is performed (Fig. 1).

Limb salvage surgery remains the mainstay of treatment for bone and soft tissue tumours. Extending limb salvage to joint salvage is possible in locally aggressive benign tumours [14] and even in malignant tumours with improved intra-operative tumour mapping and custom-guided or navigation technologies. The medical team in the operating room includes a surgeon, a surgeon’s assistant, an anaesthetist, a nurse anaesthetist, a circulating nurse, and a surgical technologist. In addition, radiation oncologists may play a key role in the operating room for carrying out brachytherapy procedures to achieve tumoricidal radiation doses for some malignant tumours. They work closely with the surgical team for optimal placement of brachytherapy catheters.

The operating room must be accessible to imaging equipment like 2D-CARM for bone procedures, 3D imaging with 3D-CARM or O-ARM, and, if feasible, navigation technology. Tumour surgery equipment for bone procedures also includes power instruments with oscillating bone-saw, electric drill system, and high-speed burr. Intramedullary reamers and third-generation bone cementing equipment with vacuum preparation and pressurization [15]. Most of these types of equipment are required on a case-to-case basis.

Since the tumours in the musculoskeletal system can vary in location as well as size, the range of instruments required for each patient varies based on the location. From distal extremity fine instruments to large pelvic or long laparotomy instruments may be required, planning the tumour resection as well as reconstruction instruments is essential. Instruments available with the functioning orthopaedic as well as oncology departments may be customized to the surgical needs of the patients.

Limb salvage surgery involves the reconstruction of soft tissue defects as well as large bone defects for functional outcomes. Recycling tumour bone, allograft bone banks, or large modular endoprosthesis are some of the options that are available with the orthopaedic onco-surgeon [16, 17]. Patient-specific 3D-printed metal implants are making a bang in orthopaedic oncology to offer custom reconstruction solutions [16, 18]. While bone reconstructions are essential for restoring limb anatomy, soft tissue reconstruction is key to restoring function and providing healthy tissue for healing [19]. Key reconstruction teams for musculoskeletal oncology include the plastic and reconstructive surgery, vascular surgery, and neurosurgery teams. With additional teams, individual team instrumentation and equipment requirements (e.g. microscope, nerve stimulator, vascular grafts) need to be planned and should be made available during the procedure. Massive surgeries in orthopaedic oncology are made possible with access to a blood bank for blood and blood product replacement in the operative as well as a post-operative course [13].

The commitment to patient care continues in the post-operative period with a robust intensive care team, physical rehabilitation, and adequate healing before the patient re-enters the team loop to receive adjuvant therapies or surveillance imaging [20].

Post-surgery, radiation oncology resumes care for planning adjuvant radiotherapy for certain tumour. This can either be in the form of brachytherapy delivered by implants placed during surgery or external beam radiotherapy delivered via linear accelerators. For brachytherapy, the patient undergoes a CT or MRI-based simulation scan, which aids a radiation oncologist in target delineation of the tumour cavity. Subsequently, a medical physicist generates a plan using advanced treatment planning systems, which helps in delivering the required dose of radiation to the target area while sparing the normal tissue. After meticulous planning, the delivery of radiation therapy is ensured by a technologist in close association with the oncologist. In the case of external beam radiotherapy, a similar procedure is executed without using the brachytherapy applicators, for treatment planning. Radiation is delivered using advanced conformal techniques like Intensity-modulated radiotherapy with image guidance utilizing x-rays, protons, or hadron therapy.

Similarly, medical oncology plans adjuvant chemotherapy in applicable cases to eliminate occult metastases. This also involves meticulous planning to instil the best available systemic agents while minimizing toxicity. Close coordination between surgery, radiation, and medical oncology is indispensable for optimal sequencing and timing of adjuvant therapies to enhance patient outcomes [11, 12].

### Leadership and the Team Process

The core team for the orthopaedic onco-surgeon remains the surgical team involved in the operating theatre and the orthopaedic/surgical wards. The role of each team member has been defined in literature and standard operating procedure manuals (Table 1).

**Table 1:** A summary of the team members and their roles is summarized

Roles in orthopaedic oncology surgery		
Site of action	Defined roles	Extended role in orthopaedic oncology
In the operating room		
Surgeon	Leads surgery	Oversees bone and soft tissue tumour resection
Anaesthetist	Manages patient anesthesia	Coordinates with the surgical team for optimal patient care
Surgical assistant	Assists the surgeon	Supports during complex tumour resections
Surgical technician	Prepares surgical instruments	Assists with specialized orthopedic oncology instruments
Scrub nurse	Manages surgical tools	Ensures sterilization and readiness of advanced tools
Nurse assistant	Provides patient support	Assists with positioning, monitoring the patient during surgery
In the ward		
Surgeon	Monitors patient post-surgery	Leads follow-up care and recovery planning
Surgical registrar	Supports post-operative care	Coordinates with a multidisciplinary team for ongoing treatment
Ward nurse	Provides nursing care	Manages patient recovery in the oncology ward
Emergency team		
Surgeon	Handles urgent complications	Specialized care for post-operative emergencies
Emergency nurse	Provides urgent care	Monitors critical cases in emergency settings

While each department has a critical role in the disease-specific outcome for the patient, the role of a multidisciplinary team is crucial to designate a team leader for orchestrating patient care. This may be a case-based individualised approach based on disease or treatment specifics. The orthopaedic onco-surgeon should take the lead in patients who are amenable to surgery. In such cases, the coordination for neoadjuvant therapy, timing of surgery, adjuvant therapy, and monitoring oncological as well as functional outcomes along with seamless patient flow should be monitored by the surgical team.

### Benefits and Disadvantages of Teamwork

There are various advantages to team collaboration in a surgical unit providing care for bone and soft tissue tumours. However, conflicting interests in a competitive work setting can affect the whole team and, more importantly, affect patient care. The combined problem-solving potential of the team is certainly more than that of an individual. As long as the professional development of an individual does not impair team cooperation, it will lead to a successful and effective team. The pursuit of a common goal leads to greater cohesion, which improves the team’s performance.

The establishment of a Musculoskeletal Oncology Unit (MSK Oncology Unit) within either the Department of Orthopaedics or the Department of Surgical Oncology presents distinct advantages and challenges. The choice of the department in which the MSK Oncology Unit resides will significantly influence its functioning, collaboration, and patient outcomes (Table 2).

**Table 2:** Below is a discussion of the pros and cons of integrating the MSK Oncology Unit into Orthopaedics as well as Surgical Oncology departments

Criteria	MSK Oncology Unit in the Department of Orthopaedics	MSK Oncology Unit in the Department of Surgical Oncology
Specialized expertise	Pros: Focus on musculoskeletal anatomy, biomechanics, and surgical interventions for functional outcomes, such as limb salvage and reconstruction	Cons: Less specialized in musculoskeletal systems, which could affect complex musculoskeletal reconstructions
	Cons: May lack broad oncology expertise, especially in chemotherapy and radiation therapies	Pros: Comprehensive cancer care approach, including chemotherapy, radiation therapy, and palliative care
Surgical resources and support	Pros: Well-established infrastructure for musculoskeletal surgeries and specialized equipment	Cons: May lack specialized tools and resources for musculoskeletal-specific surgeries
	Cons: Less access to broader oncological treatments (e.g. chemotherapy and radiation therapy) within the department	Pros: Access to a robust cancer care infrastructure, including oncologists, radiologists, and multidisciplinary tumour boards
Continuity of care	Pros: Strong focus on long-term follow-up and rehabilitation, ensuring functional recovery	Cons: May lack emphasis on rehabilitation, potentially leading to gaps in post-operative recovery
	Cons: Can become isolated from other oncology disciplines, leading to fragmented care in complex cases	Pros: Holistic, multidisciplinary approach ensures seamless coordination of all cancer treatments and supportive care
Multidisciplinary collaboration	Cons: Orthopaedic departments may be siloed, reducing integration with broader oncology specialties	Pros: Established collaboration between surgical oncologists, medical oncologists, radiation oncologists, and pathologists, ensuring coordinated care
Focus on oncological treatment	Cons: May prioritize surgical interventions over integrated cancer treatments like chemotherapy and radiotherapy	Pros: Ensures a balanced approach to surgical, medical, and radiation treatments, offering a comprehensive cancer treatment plan
Rehabilitation and functional restoration	Pros: Close relationship with physical therapists and rehabilitation teams, crucial for musculoskeletal recovery	Cons: Less emphasis on rehabilitation and functional outcomes, with less interaction with physical therapy teams
Cancer-specific expertise	Cons: Limited exposure to specialized oncological treatments such as chemotherapy and radiation	Pros: Strong focus on comprehensive cancer management, providing expertise across all oncological domains (medical, surgical, radiation)
Resource allocation	Pros: Focused on musculoskeletal surgeries, ensuring resources for complex musculoskeletal cases	Cons: Potential for limited resources for MSK oncology cases due to broad cancer focus
Integration of advanced technology	Pros: Access to specialized musculoskeletal technologies (e.g. navigation systems, 3D-printed implants)	Cons: May lack cutting-edge musculoskeletal-specific technology, such as advanced imaging and navigation tools
Post-surgical care and recovery	Pros: In-depth expertise in managing complex musculoskeletal post-surgical care, including rehabilitation	Cons: Less focus on musculoskeletal recovery, which could delay rehabilitation and return to function
Overall approach to cancer care	Cons: Might be less comprehensive in terms of multidisciplinary cancer care	Pros: A holistic, cancer-focused approach integrates all aspects of oncological treatment, improving patient outcomes

### Audit and Measurement of Outcomes

The oncological and functional outcomes are essential for patients with bone or soft tissue tumours [21]. To assess these outcomes, an audit of the unit is a must. Literature shows that in practice, surgical team audits are insufficiently and inconsistently measured. The orthopaedic oncologist must establish a robust list of parameters that are deemed useful for measuring outcomes and can always fall back on national registries or the musculoskeletal tumour registry to record adequate and consistent data. Along with records, it is essential to audit the data, analyse the scope for improvement, and compare outcomes with the literature [22].

### Education

Comprehensive education is critical for developing expertise in the subspecialty of orthopaedic oncology. Residents [5] and fellows undergo rigorous training under experienced faculty mentors [7]. This includes participation in clinics, operating rooms, tumour boards, and multidisciplinary conferences. Hands-on surgical skills labs allow the practice of techniques like limb salvage resection, implant fixation, and wound closures using simulation models and cadavers [13]. Relevant coursework dives into tumour biology, medical imaging, radiation/medical oncology principles, musculoskeletal pathology, surgical oncology, and reconstruction methods. Multidisciplinary tumour boards and combined clinics promote interaction with radiology, pathology, and allied oncology specialists, providing perspectives from related disciplines [6, 11, 12]. Education on emerging treatment protocols and adjuvant therapies ensures that residents are well versed in current standards of care. Communication skill training in counselling and rehabilitation is vital for holistic patient care. Research exposure through clinical trials, translational projects, and collaborations with basic scientists instils evidence-based thinking [23]. Participation in academic activities like journal clubs, lectures, and conferences keeps doctors updated on the latest advancements. Awareness programs among orthopaedic and general surgeons regarding the correct approach to bone/soft tissue tumours need to be highlighted.

### Research

A thriving research program is integral for the academic orthopaedic oncology department. Clinical trials assessing novel techniques, implants, and adjuvant therapies foster evidence-based care. Institutional databases and cancer registries enable analysis of long-term patient outcomes. Translational research applies insights from genomics, proteomics, and basic sciences to address clinical challenges. Collaborations with engineers, immunologists, and oncology researchers facilitate studying emerging solutions like nanotechnology, 3D printing [16], and immunotherapies. Health services research improves quality, safety, access, patient experience, and cost-effectiveness [24]. Dissemination through publications, presentations, and exchanges expands academic influence. The overarching goal is to apply research to improve clinical care and outcomes for patients.

### Public Health

Advancing public health is a key priority in orthopaedic oncology to improve access and delivery of care on a broader scale. Establishing centres of excellence with specialized expertise and resources allows for the concentration of care for better outcomes. Outreach programs focused on education, early diagnosis [25], and screening facilitate detection at more treatable stages [26], especially in underserved communities. Telemedicine [27] and mobile health technology can help to provide expert care to remote areas through virtual tumour boards [28], consultations, and follow-ups. Patient support groups [20] play a key role in raising awareness and offering help to patients and families. Organizations dedicated to sarcoma support are instrumental in increasing public knowledge about this condition, organizing fundraising events to further research efforts, and providing valuable emotional and practical assistance to affected individuals [29]. Legislative efforts are needed to make care equitable and affordable through policy changes. A broad public health perspective is thus integral to driving progress in prevention [30], access, experience, and outcomes for patients with musculoskeletal tumours.

## Conclusion

The establishment of a Musculoskeletal Oncology Unit within a tertiary cancer care setting marks a significant advancement in the management of musculoskeletal malignancies. By fostering a collaborative environment among diverse medical professionals—including orthopaedic surgeons, radiologists, pathologists, medical and radiation oncologists, and rehabilitation specialists—we ensure that patients receive the holistic, multidisciplinary care they require. This integrative approach not only enhances the delivery of services but also optimizes patient outcomes, reflecting the necessity of teamwork in this specialized field. As we reflect on our experience, it is clear that the notion of a free-standing orthopaedic oncologist is a myth; true success in managing musculoskeletal tumours relies on a cohesive team and the integration of cutting-edge medical technologies. Moving forward, we advocate for continued investment in collaborative models, comprehensive training programs, and research initiatives that will further elevate the standard of care in orthopaedic oncology, ultimately benefiting our patients and advancing public health efforts in this critical area.

## Data Availability

Data available on reasonable request.
